# Cost-Effectiveness Analysis of Routine Childhood Immunization with 20-Valent versus 15-Valent Pneumococcal Conjugate Vaccines in Germany

**DOI:** 10.3390/vaccines12091045

**Published:** 2024-09-12

**Authors:** Min Huang, Jessica P. Weaver, Elamin Elbasha, Thomas Weiss, Natalie Banniettis, Kristen Feemster, Meghan White, Matthew S. Kelly

**Affiliations:** 1 Merck Research Laboratory, Merck & Co., Inc., Rahway, NJ 07065, USA; jessica.weaver@merck.com (J.P.W.); elamin_elbasha@merck.com (E.E.); thomas_weiss@merck.com (T.W.); natalie.banniettis@merck.com (N.B.); kristen.feemster@merck.com (K.F.); meghan.white3@merck.com (M.W.); 2 Division of Pediatric Infectious Diseases, Duke University School of Medicine, Durham, NC 27710, USA; matthew.kelly@duke.edu

**Keywords:** cost-effectiveness, PCV15, V114, PCV20, pneumococcal diseases, childhood immunization, Germany

## Abstract

This study aimed to evaluate the cost-effectiveness of routine childhood immunization with the 20-valent pneumococcal conjugate vaccine (PCV20) in a four-dose regimen (3 + 1 schedule) versus the 15-valent PCV (PCV15/V114) in a three-dose regimen (2 + 1) in Germany. The study utilized a decision-analytic Markov model to estimate lifetime costs and effectiveness outcomes for a single birth cohort in Germany. The model tracked the incidence of acute pneumococcal infections and long-term pneumococcal meningitis sequelae for both vaccination strategies. The vaccine effectiveness data were derived from published clinical trials and observational studies of PCV7 and PCV13. Indirect effects, such as herd protection and serotype replacement, were included in the model. The model adopted a societal perspective, including direct medical, direct non-medical, and indirect costs. Scenario and sensitivity analyses were performed. In the base case, PCV20 prevented more pneumococcal disease cases and deaths, with an expected gain of 96 quality-adjusted life years (QALYs) compared to V114. However, PCV20 was associated with a total incremental cost of EUR 48,358,424, resulting in an incremental cost-effectiveness ratio (ICER) of EUR 503,620/QALY. Most of the scenario and sensitivity analyses estimated that the ICER for PCV20 exceeded EUR 150,000/QALY. Routine childhood immunization with PCV20 instead of V114 may not be an economically efficient use of healthcare resources in Germany.

## 1. Introduction

*Streptococcus pneumoniae* (the pneumococcus) is a leading cause of infectious disease morbidity and mortality worldwide [1,2]. According to the 2021 Global Burden of Diseases study, *S. pneumoniae* accounted for more cases and deaths related to lower respiratory tract infection than any other etiology [2]. It is also among the top five leading pathogens for deaths associated with antibiotic resistance globally [3]. Invasive pneumococcal disease (IPD) represents a group of diseases in which *S. pneumoniae* is isolated or detected from blood or another normally sterile site, and includes meningitis, bacteremia without a focus, and bacteremic pneumonia [4]. IPD is associated with a high case fatality rate and can lead to debilitating long-term sequelae, including permanent neurological deficits and hearing loss that occur frequently after recovery from pneumococcal meningitis [5,6]. Non-invasive pneumococcal diseases, including non-bacteremic pneumococcal pneumonia (NBPP) and acute otitis media (AOM), have lower case fatality rates but occur far more frequently than IPD [4,7].

Pneumococcal vaccines were developed to protect against *S. pneumoniae* infections caused by common disease-causing serotypes. In Germany, the seven-valent pneumococcal conjugate vaccine (PCV7) was licensed in 2001, and recommended for use in children less than two years of age by the German Standing Committee on Vaccination (STIKO) in July 2006. The 10-valent PCV (PCV10) and 13-valent PCV (PCV13) were licensed in Germany in 2009, and replaced PCV7 in the routine childhood immunization schedule. Initially, PCV10 and PCV13 were given as a three-dose primary infant series followed by a toddler dose (3 + 1 schedule), but administration in a two-dose primary infant series followed by a toddler dose (2 + 1 schedule) was recommended in 2015 [8,9]. The administration of these vaccines in a 2 + 1 schedule has been shown to have similar vaccine effectiveness (VE) while being more cost-effective than administration in a 3 + 1 schedule, and it has been endorsed by the World Health Organization [10].

The introduction of PCVs in Germany substantially reduced the incidence of IPD in children, but also led to marked shifts in the serotype epidemiology of these infections [8,11]. The proportion of IPD isolates caused by PCV13 serotypes declined in children (<16 years of age) from 2010 to 2014, except for serotype 3, for which the proportion increased following both the introduction of PCV7 and the later switch to PCV10 and PCV13 [8]. Similar reductions in the proportion of IPD cases caused by PCV13 serotypes were observed among adults due to herd protection [8]. However, the incidence of IPD cases caused by non-PCV13 serotypes among children < 16 years of age increased substantially from 2011–2012 to 2015–2016, primarily as a result of serotype replacement [11].

Higher-valency PCVs were recently recommended for use in Europe to provide improved protection against pneumococcal disease. In 2022, the European Medicines Agency (EMA) approved the 15-valent PCV (PCV15 or V114) for use in a 2 + 1 or 3 + 1 schedule for the prevention of pneumococcal disease in children [12]. V114 contains the 13 serotypes in PCV13 and the additional serotypes, 22F and 33F. Compared to PCV13, V114 (2 + 1) results in non-inferior serum antibody responses to the 13 shared serotypes and superior serum antibody responses for serotypes 22F and 33F [13,14]. Since 2023, V114 has been recommended by STIKO as an alternative to PCV13 for routine childhood immunization, administered as a 2 + 1 schedule for full-term infants [15].

The twenty-valent PCV (PCV20), which contains five additional serotypes not included in V114 (8, 10A, 11A, 12F, and 15B), was approved by the EMA for the vaccination of children in 2024 [16]. However, in contrast to PCV13 and V114, which were approved by the EMA for use in both 3 + 1 and 2 + 1 schedules, PCV20 is approved for use only in a 3 + 1 schedule. This approval was granted on the basis of data from two pivotal phase 3, randomized controlled trials that compared the safety and immunogenicity of PCV20 and PCV13 in children [17]. In the first trial (B7471012), which compared PCV20 and PCV13 administered in a 2 + 1 schedule, serum antibody responses among children after receiving the PCV20 primary infant series did not reach the statistical non-inferiority criterion for nine of the thirteen serotypes shared across these vaccines or for two of the seven additional serotypes contained within PCV20 [17]. In the second trial (B7471011), in which these vaccines were administered in a 3 + 1 schedule, serum antibody responses among children after receiving PCV20 primary infant series did not reach the co-primary endpoint of non-inferiority for five of thirteen shared serotypes and one additional PCV20 serotype [17]. After considering these immunogenicity data, the Committee for Medicinal Products for Human Use within the EMA raised concerns over the effectiveness of PCV20 administered in a 2 + 1 schedule, and only recommended its use in a 3 + 1 schedule [17].

V114 and PCV13 are currently reimbursed in Germany. In light of the EMA approval of PCV20, it is valuable to assess its health economic value as a potential candidate for routine childhood immunization. The current study aimed to evaluate the cost-effectiveness of routine childhood immunization with PCV20 (3 + 1) and V114 (2 + 1) from a societal perspective in Germany. The results from this analysis have the potential to inform public health and clinical decisions regarding routine childhood immunization with PCVs in Germany.

## 2. Methods

This cost-effectiveness analysis (CEA) compared two approaches for routine childhood immunization with PCVs in Germany: a potential future strategy with PCV20 administered in a 3 + 1 schedule versus a current strategy recommended by STIKO with V114 administered in a 2 + 1 schedule. The 3 + 1 schedule of PCV20 used in the model was based on the EMA-approved indication for PCV20 in the Summary of Product Characteristics [16], with primary infant series doses administered at approximately 2, 4, and 6 months of age and the toddler dose administered at the beginning of the second year of life. The timing of doses of V114 also followed the EMA Summary of Product Characteristics, with primary series doses administered at 2 and 4 months of age, followed by the toddler dose at the beginning of the second year of life [12]. The model did not consider partial completion of the primary infant series or mixed use of PCV20 and V114.

### 2.1. Model Overview

**Figure 1:**
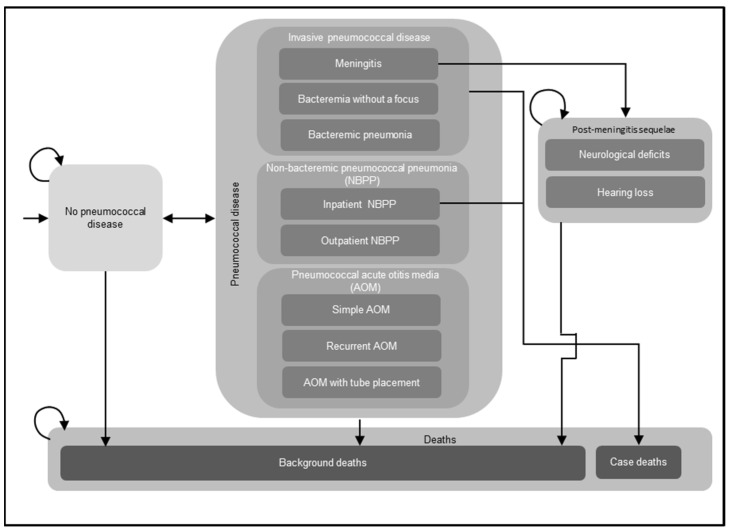
Decision-analytical Markov model structure. Abbreviations: NBPP = non-bacteremic pneumococcal pneumonia; AOM = acute otitis media. Notes: This figure illustrates the potential health states and acute diseases that a birth cohort might experience in their lifetime. The model assumed that a single birth cohort in Germany entered the model without pneumococcal disease and might develop invasive pneumococcal disease (IPD), NBPP, and pneumococcal AOM, or transition to other health states, i.e., post-meningitis sequelae (PMS), including neurological deficits, hearing loss, and death. Individuals who developed PMS remained in this health state until death and were at risk of developing non-meningitis IPD, NBPP, and pneumococcal AOM in subsequent model cycles. In addition, the model assumed that individuals with IPD and inpatient NBPP experienced excess mortality, while individuals who developed other pneumococcal diseases (outpatient NBPP and AOM) were considered to have the same age-specific mortality as the general population. The curved arrows indicate that individuals might remain in the same health state in the next model cycle.

A decision-analytic Markov model was utilized to assess the costs and effectiveness outcomes of routine childhood immunization with PCVs (Figure 1). The Markov model structure has been used extensively in previously published CEAs of childhood immunization with PCVs, including several prior CEAs conducted in Germany [18,19,20,21]. For the current analysis, a single birth cohort in Germany entered the model, and individuals were followed over their lifetimes for the development of pneumococcal disease, including IPD, NBPP, and pneumococcal AOM; post-meningitis sequelae (PMS), including neurological deficits and hearing loss; and death. Newborns entered the model without pneumococcal disease at cycle 0 and either remained without pneumococcal disease, experienced acute episodes of pneumococcal disease, or transitioned to the PMS or death health states in any given model cycle spanning one year. All acute episodes of pneumococcal disease were assumed to resolve within one year and incur short-term resource utilization, costs, and decrements in quality-adjusted life years (QALYs) during the cycle in which these infections occurred. Individuals who developed PMS remained in this health state until death and were at risk of developing non-meningitis IPD, NBPP, and pneumococcal AOM in subsequent model cycles. The model assumed that individuals with IPD and inpatient NBPP experienced excess mortality, while individuals who developed other pneumococcal diseases (outpatient NBPP and AOM) were considered to have the same age-specific mortality as the general population. The model incorporated both direct and indirect effects (i.e., herd protection and serotype replacement) of PCVs on pneumococcal disease.

The base-case analysis adopted a societal perspective, and considered direct medical, direct non-medical, and indirect costs. Clinical outcomes, including the numbers of IPD, NBPP, pneumococcal AOM, and PMS cases prevented and the numbers of deaths from IPD and NBPP averted were estimated. Cost outcomes included each cost component, as well as the total costs from the societal perspective. Incremental cost-effectiveness ratios (ICERs) were estimated as the incremental cost per QALY gained and incremental cost per life year gained. Following the Institute for Quality and Efficiency in Health Care recommendations, an annual discount rate of 3% was applied to cost outcomes, QALYs, and life years [22].

### 2.2. Model Inputs

#### 2.2.1. Population Inputs

The target population consisted of a birth cohort of 738,819 infants, corresponding to the number of live births in Germany in 2022, as reported by the Federal Statistical Office [23]. The age-specific background mortality rate, which was applied to all individuals in the model, was similarly obtained from data published by the Federal Statistical Office of Germany [24].

#### 2.2.2. Epidemiological Inputs

Annual incidence rates for pneumococcal diseases were derived from published research (Table 1). Annual incidence rates for IPD and all-cause pneumonia were obtained from two retrospective cohort studies that analyzed data from the InGef database (Institute for Applied Health Research Berlin), which contains longitudinal claims data from individuals throughout Germany [25,26]. For NBPP, annual incidence rates were estimated by multiplying all-cause pneumonia incidences by the proportion of pneumonia cases attributable to *S. pneumoniae*, which was estimated to be 9.2% based on data from a retrospective study of >3000 adults in Germany and 10 other European countries [27]. The proportion was applied to both children and adults in the model due to the scarcity of data available for children. Similarly, annual incidence rates of pneumococcal AOM—including simple AOM, recurrent AOM, and AOM with tympanostomy tube placement—were estimated by multiplying the annual incidence rates for all-cause AOM reported in an observational study in Germany [28] by the percentage of AOM cases attributed to *S. pneumoniae* (6.61%) in a multi-center study of AOM among German children [29]. Case fatality rates for IPD were obtained from a national surveillance study of IPD in Germany [30] and a study that analyzed data from the German InGef database [25]. Case fatality rates for inpatient NBPP were obtained from the same studies from which the incidence rates of IPD were estimated using the German InGef database [25,26]. Finally, the model assumed that 5.7% and 8.3% of meningitis cases would result in neurological deficits and hearing loss, respectively, based on data reported in the aforementioned German IPD surveillance study [30].

The age-specific proportions of pneumococcal disease cases caused by serotypes unique to PCV20 (PCV20-non-V114) are presented in Table 2. For IPD, inputs were obtained from an IPD surveillance report provided by van der Linden and colleagues, using the most recent time period for which data were available (2022–2023) [31]. The same serotype distribution was applied to NBPP due to lack of data for this disease. For pneumococcal AOM, the serotype distribution was obtained from an observational study conducted in Germany from 2012–2015 [29], as these were the most recent country-specific data available.

#### 2.2.3. Vaccine Coverage Rates

Vaccine coverage rates were sourced from a recently published study that used a claims database to determine childhood vaccination rates in Germany before and after the change in administration of PCVs from a 3 + 1 schedule to a 2 + 1 schedule [32]. Specifically, coverage rates for PCV20 (3 + 1) were estimated to be 86% for completion of the primary infant series and 68% for the toddler dose, while the corresponding coverage rates for V114 (2 + 1) were estimated to be 89% and 76%, respectively [32].

#### 2.2.4. Vaccine Effectiveness

##### Direct Effects Estimation

Direct effects were modeled as serotype-specific VE (Table 3), which was defined as the percentage reduction in serotype-specific pneumococcal disease incidence rates relative to the period before the specific vaccination strategy was implemented. The VE inputs for V114 and PCV20 were derived from the VE of PCV7 or PCV13 estimated from relevant clinical trials or observational studies. Post-primary series (PPS) VE and post-toddler dose (PTD) VE were estimated separately in this model. A key assumption for PPS VE was that there were reductions in VE for the serotypes in PCV20 that did not meet the pre-specified non-inferiority criteria compared to PCV13 in the phase 3 trial of PCV (3 + 1) [17]. For PTD VE, the model assumed that V114 and PCV20 had the same serotype-specific VE for the shared serotypes. The estimation of VE inputs and specific assumptions used to estimate PPS and PTD VE are described below. 

###### Direct-Effects Inputs

The serotype-specific VE of V114 against IPD was derived from an observational study on use of PCV10 and PCV13 in European children [33]. The VE of V114 was assumed to be the same as PCV13 for the shared serotypes, and the overall VE of PCV13 against the thirteen vaccine serotypes was used for the two additional serotypes included in V114 (i.e., 22F and 33F). For PCV20, the VE for PCV7 serotypes was derived from a case–control study of PCV7 (3 + 1) [34], and the VE for the six PCV13-non-PCV7 serotypes was obtained from a case–control study of PCV13 (3 + 1) [35]. Similar to V114, the overall VE of PCV13 against the vaccine serotypes was applied to the PCV20-non-PCV13 serotypes.

The serotype-specific VE of V114 and PCV20 against inpatient and outpatient NBPP was obtained from a surveillance study conducted in southern Israel [36], which estimated the VE of PCV13 against community-acquired pneumonia with concurrent PCV13 serotype carriage to be 77.0% among children who received PCV13 (2 + 1). The same VE was assumed for all serotypes.

For simple and recurrent pneumococcal AOM, the VE for both V114 and PCV20 against PCV7 serotypes was obtained from a clinical trial of PCV7 (3 + 1) that was conducted in Finland [37], while the VE for the six PCV13-non-PCV7 serotypes was sourced from an observational study of PCV13 (3 + 1) conducted in the United States (US) [38]. VE for additional serotypes included in V114 or PCV20 but not PCV13 was assumed to be the same as the overall VE of PCV7 against all seven vaccine serotypes [37]. For all serotypes in V114 or PCV20, VE against pneumococcal AOM requiring tympanostomy tube placement was estimated to be 68.8%. This value was derived from the 20.3% VE against all-cause AOM requiring tympanostomy tube placement reported in a clinical trial of PCV7 (3 + 1) [39], adjusted for the estimated proportion of all-cause AOM attributable to *S. pneumoniae* (44%) [40] and the proportion of pneumococcal pneumonia cases accounted for by PCV7 serotypes in a study conducted in the US prior to introduction of PCV (67.1%) [41].

###### Estimations of PPS VE and PTD VE for V114 and PCV20

The PPS VE inputs for V114 were the same as those described above. The reduced VE for PCV20 was applied to the six serotypes that failed to achieve the co-primary objective of non-inferiority based on the percentage of participants who reached a pre-specified PPS serum serotype-specific immunoglobulin G (IgG) level in the phase 3 trial of PCV20 administered in a 3 + 1 schedule (B7471011) [17]. Specifically, a 50% relative reduction in VE was assumed for serotype 3, while a 25% relative reduction in VE was assumed for serotypes 1, 4, 9V, 12F, and 23F. A higher percentage of VE reduction was assumed for serotype 3 because the 95% confidence intervals for the proportion of individuals reaching the pre-specified serum IgG level was entirely below the non-inferiority margin of −10% in the co-primary objective [17]. The reduced VE for these serotypes was applied for all pneumococcal disease categories.

For PTD VE, V114 and PCV20 were assumed to have equivalent serotype-specific VE for the shared serotypes on the basis of immunogenicity data available for PCV13 and V114. These data showed PTD-serotype-specific antibody levels were higher than PPS antibody levels, probably as a result of immune system maturation during infancy and immune priming from infant vaccine doses [42,43]. In addition, a recent study assessing the VE of PCV13 against IPD using cases from the Active Bacterial Core surveillance through the US Centers for Disease Control and Prevention (CDC) reported comparable PTD VE with 2 + 1 and 3 + 1 schedules [44]. Because the estimated serotype-specific VE inputs could differ between V114 and PCV20, the larger serotype-specific PPS VE estimated for these two vaccines was used as the PTD VE input for both vaccines for each shared serotype.

##### Vaccine Effectiveness Onset and Waning

For both PCV20 (3 + 1) and V114 (2 + 1), the first two vaccine doses would be administered at 2 and 4 months of age for routine childhood vaccination in Germany. Based on data regarding the VE of PCV7 for IPD during the course of infancy [34], we assumed that the first dose of these vaccines conferred 76.8% of the PPS VE, with the full PPS VE being realized after receipt of the second dose. Therefore, for both PCV20 and V114, vaccination with the primary infant series was assumed to confer 79.5% of the estimated PPS VE during the first year of life. For children who only completed the primary infant series, the full PPS VE was applied from the 2nd to the 5th year of life, followed by a linear reduction to 0% over the next five years. For children who additionally received the toddler dose, the model assumed the full PTD VE during the first five years after the last dose and a linear decline to 0% over the next 10 years [45].

#### 2.2.5. Indirect Effects

Indirect effects of the vaccines, including herd protection and serotype replacement, were applied only to IPD. Indirect effects were not modeled for NBPP or pneumococcal AOM due to a lack of evidence supporting consistent trends in indirect effects of PCVs on these diseases in Germany. Specifically, a multi-center prospective cohort study of community-acquired pneumonia conducted among adults in Germany observed that the proportion of cases caused by PCV13 serotypes remained stable following the replacement of PCV7 with PCV10 and PCV13 for routine childhood immunization in Germany [46]. Additionally, there are a lack of data supporting indirect effects of PCVs on pneumococcal AOM in Germany.

Indirect effects of childhood immunization with PCVs on IPD were modeled as a relative reduction or increase in incidence resulting from universal use of PCVs. In the base case, herd protection for new vaccine serotypes and serotype replacement by non-vaccine serotypes were estimated from a German surveillance study [8] and applied for both V114 and PCV20. The maximum herd protection effect (70.0% for children 0–15 years of age and 42.2% for individuals 16 years of age or older) was calculated based on the reduction in the number of IPD cases caused by PCV13-non-PCV7 serotypes observed in vaccine-ineligible age groups in the years following introduction of PCV10 and PCV13 (2010–2014) [8] and was applied to each new vaccine serotype (V114: 22F, 33F; PCV20: 22F, 33F, 8, 10A, 11A, 12F, 15B). The estimated maximum reduction in overall incidence of IPD due to herd protection effect ranged from 2.1–4.0% for V114 and 5.7–15.0% for PCV20 across different age groups. The maximum serotype replacement effect of PCV13 (29.6% in children 0–15 years of age, 39.3% in individuals 16 years of age or older) was derived from increases in the number of IPD cases caused by non-PCV13 serotypes in Germany between 2010 and 2014 [8]. The effect was assumed to depend on the ratio of the proportion of infections caused by new serotypes contained within a vaccine to the proportion of infections caused by non-vaccine serotypes. Therefore, it was adjusted based on the ratio between the incidence of IPD caused by PCV20/V114-non-PCV13 serotypes and the incidence of IPD caused by non-PCV20/V114 when estimating the serotype replacement effect for PCV20 and V114 [31]. The estimated maximum increases in the overall incidence of IPD due to serotype replacement effect ranged from 1.1–2.7% for V114 and 2.4–9.1% for PCV20 across different age groups. For both herd protection and serotype replacement, the maximum indirect effects were assumed to have been reached five years after vaccine introduction and were applied to the entire modeled cohort, including both vaccinated and unvaccinated individuals.

#### 2.2.6. Utility Inputs

QALY decrements associated with each episode of pneumococcal disease were sourced from previously published CEAs of PCVs (Table 4). Specifically, the QALY decrements associated with IPD, NBPP, and pneumococcal AOM in children less than 18 years of age were obtained from a CEA of PCV7 conducted in the US [47]. The QALY decrements associated with IPD and NBPP in adults 16 years of age or older were sourced from a CEA conducted in the Netherlands [48]. The utility values used for PMS health states—0.68 for neurological deficits and 0.73 for hearing loss—were also sourced from the CEA conducted in the US [47], and were applied to all individuals in these health states irrespective of age. Individuals without pneumococcal disease or PMS were assumed to have the same utility values as the general population in Germany [49].

#### 2.2.7. Cost Inputs

The base case considered direct and indirect costs, which included vaccine acquisition and administration costs, direct and indirect costs associated with the treatment of pneumococcal diseases, and direct and indirect costs associated with PMS and premature death (Table 5). All costs were adjusted to 2023 EUR using the healthcare consumer price index [50].

##### Vaccine Acquisition and Administration Costs

Prices per dose of PCV20 and V114 were obtained from publicly available data [51]. Vaccine administration costs were estimated at EUR 8 per dose, which was the average reimbursement fee from a sample of vaccination agreements between the German statutory health insurance (SHI) system and the National Association of Statutory Health Insurance Physicians. It was assumed that PCVs were administered at the same time as other vaccines and that no additional visit costs were incurred.

##### Costs Associated with Pneumococcal Disease

Direct medical costs for treating each episode of IPD, NBPP, and pneumococcal AOM were estimated from published studies in Germany and differed for individuals less than 16 years of age and those 16 years of age or older [25,52].

Indirect costs had two components: productivity loss due to disease treatment among patients or caregivers and premature death among patients. Model inputs for the indirect costs associated with productivity loss were estimated using the missed workdays for each pneumococcal disease episode and the average annual earnings within each age group in Germany. The number of missed workdays was derived from the inputs used by an international study to estimate indirect costs associated with productivity loss among caregivers for children with pneumococcal disease in Germany and 12 other countries [53]. The annual average earnings for each age group were calculated by multiplying the average annual gross earnings by the labor force participation rate for each age group using data from the Federal Statistical Office of Germany [54,55]. Indirect costs associated with premature death among individuals with IPD or inpatient NBPP represented lost earnings estimated from the annual average earnings for each age group, age at premature death, and the expected life expectancy in Germany [24,54,55].

##### Costs Associated with PMS

For individuals with PMS, annual direct medical and non-medical costs associated with neurological deficits and hearing loss were obtained from a multi-country study in which the annual direct costs of PMS in Germany were derived from existing research [56], and were applied to individuals with PMS regardless of age. In addition, the model assumed that individuals with PMS were not employed. Indirect costs associated with productivity loss due to PMS were estimated based on the average annual earnings for each age group in Germany and applied only to individuals 16 years of age or older [54,55].

### 2.3. Scenario and Sensitivity Analyses

Scenario analyses were performed with alternative assumptions and inputs regarding the study population, time horizon, model perspective, direct and indirect vaccine effects, epidemiological inputs, and health utilities. Specifically, a 10-year time horizon, a target population including the entire German population, and a healthcare sector perspective were evaluated in scenario analyses. Moreover, given the uncertainties regarding vaccines’ direct effects, particularly the assumptions made regarding the VE of PCV20, a scenario analysis was conducted assuming no reduction in VE for the serotypes that did not meet the non-inferiority criterion in the phase 3 trial of PCV20 administered in a 3 + 1 schedule (B7471011). Additional scenario analyses used estimates for VE against IPD from two modeling analyses based on immunogenicity data for PCV20 and V114 [57,58], and a VE against NBPP estimated from an observational study conducted in China [59] (Supplementary Table S1). The model considered a scenario with alternative inputs and assumptions for indirect effects from a CEA conducted in the USA, which applied a 7.8% annual reduction in the incidence of IPD for the herd protection effect and did not model serotype replacement. These inputs and assumptions regarding herd protection effect and serotype replacement were based on data from Active Bacterial Core surveillance conducted by the US CDC [60]. Additionally, a scenario assuming re-emergence of IPD caused by serotypes that did not meet the non-inferiority criterion in the PCV20 (3 + 1) clinical trial was also performed to evaluate the potential long-term impact of inferior serum antibody response of PCV20. Other scenario analyses used estimates of the serotype distribution for IPD and NBPP from data collected prior to the COVID-19 pandemic in 2018–2019 (Supplementary Table S2) [31], applied alternative proportions of pneumonia and AOM cases attributable to *S. pneumoniae* using data presented at a US CDC Advisory Committee on Immunization Practices (ACIP) meeting in 2023 [61], included an alternative assumption regarding vaccine effect waning, used QALY decrement inputs sourced from a recent pooled analysis (Supplementary Table S3) [62], or assumed the vaccine coverage rates for PCV20 (3 + 1) were the same as the ones for V114 (2 + 1).

One-way sensitivity analyses and a probabilistic sensitivity analysis (PSA) were conducted to evaluate various input parameters, with the exception of the vaccine prices. One-way sensitivity analyses varied one model input at a time to assess the sensitivity of the model results to each input parameter. The PSA tested the robustness of the model with respect to uncertainty in all input parameters, wherein a theoretical probability distribution was assigned to each parameter and a Monte Carlo simulation with 1000 iterations was performed by varying all parameters simultaneously.

## 3. Results

### 3.1. Base Case

In the base case (Table 6), compared to V114 and over the lifetime of the birth cohort, PCV20 (3 + 1) was projected to result in 86 fewer cases of IPD, 672 fewer cases of NBPP, 2219 fewer cases of pneumococcal AOM, and three fewer cases of PMS. Furthermore, PCV20 was projected to prevent 10 more IPD-related deaths and to result in an additional 96 QALYs and 75 life years. However, PCV20 (3 + 1) was also associated with substantially higher costs. Compared to V114 (2 + 1), use of PCV20 (3 + 1) resulted in a total incremental cost of EUR48,358,424 over the lifetime of the birth cohort. The incremental costs were primarily attributable to higher vaccine acquisition and administration costs associated with PCV20 compared to V114, which totaled EUR 49,612,973. Lower direct medical costs and indirect costs associated with pneumococcal disease were observed with PCV20, but these did not offset the additional vaccine costs associated with the use of PCV20. Combining costs and effectiveness, the ICERs of PCV20 (3 + 1) compared to V114 (2 + 1) were EUR 503,620 per QALY gained and EUR 648,546 per life year gained.

### 3.2. Scenario and Sensitivity Analyses

The results from the scenario analyses (Table 7) showed that the ICERs for PCV20 compared to V114 ranged from EUR 183,006 per QALY gained to PCV20 being dominated by V114 (i.e., lower QALYs and higher costs). The lowest ICER for PCV20 corresponded to the scenario in which the entire German population was considered in the model instead of a single birth cohort. PCV20 was dominated by V114, with the assumption of the re-emergence of IPD caused by the specific serotypes in PCV13 for which PCV20 was assumed to have reduced VE (serotypes 1, 4, 9V, and 23F). The ICER for PCV20 also showed considerable sensitivity to the time horizon, IPD serotype distribution, and inputs for indirect effects. Specifically, reducing the time horizon to 10 years increased the ICER for PCV20 to EUR 2,672,457 per QALY gained, while the use of IPD serotype data from Germany prior to the COVID-19 pandemic (2018–2019) [31] reduced the ICER to EUR 210,403 per QALY. The ICER for PCV20 declined to EUR 295,248 per QALY gained, assuming smaller herd protection effects and no serotype replacement based on a CEA conducted in the US [60]. Furthermore, using the estimated proportions of pneumonia and AOM cases attributable to *S. pneumoniae* from the 2023 CDC ACIP meeting presentation [61] had a moderate impact on the ICER for PCV20, reducing it to EUR 424,805 per QALY. Assuming no VE reductions for PCV20 also had a moderate impact on the ICER for PCV20 (EUR 412,159 per QALY), while using alternative VE inputs for IPD and NBPP resulted in a higher ICER for PCV20 (EUR 606,835 per QALY). The waning of vaccine effects, alternative QALY decrement inputs, taking a healthcare sector perspective or applying the same vaccine coverage rates for PCV20 and V114 had relatively small impacts on the ICER for PCV20 compared to V114.

**Figure 2:**
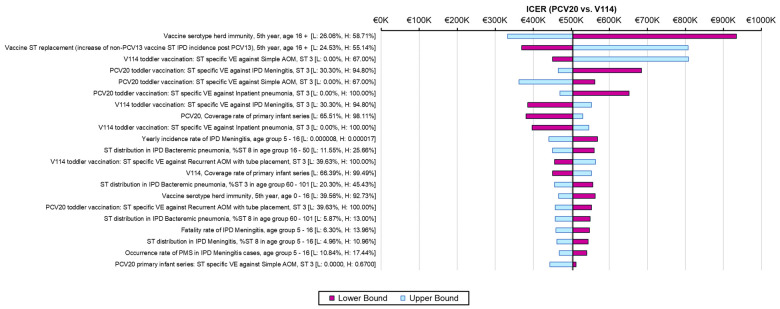
Results of one-way sensitivity analyses. Abbreviations: ICER = incremental cost-effectiveness ratio; PCV20 = 20-valent pneumococcal conjugate vaccine; V114 = 15-valent pneumococcal conjugate vaccine; QALY = quality-adjusted life year; VE = vaccine effectiveness; IPD = invasive pneumococcal disease; AOM = acute otitis media; ST = serotype. Notes: Tornado diagram depicting the results of one-way sensitivity analyses. The pink and light-blue bars show changes in the ICER for PCV20 (i.e., incremental costs per QALY gained) compared to V114 from the base-case analysis (black line) when the lower or upper values of an input were used, with all other model inputs being held constant.

**Figure 3:**
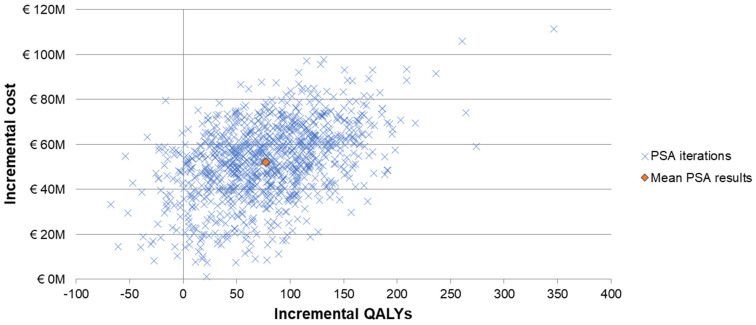
Scattered plot of probabilistic sensitivity analysis comparing PCV20 (3 + 1 schedule) to V114 (2 + 1). Abbreviations: QALY = quality-adjusted life year; PCV20 = 20-valent pneumococcal conjugate vaccine; V114 = 15-valent pneumococcal conjugate vaccine; PSA = probabilistic sensitivity analysis. Notes: The PSA tested the robustness of the model with respect to uncertainty in all input parameters with the exception of the vaccine prices, wherein a theoretical probability distribution was assigned to each parameter and a Monte Carlo simulation with 1000 iterations was performed by varying all parameters simultaneously. Each “x” represents the results from one iteration. The orange diamond depicts the mean PSA results from the 1000 iterations.

**Figure 4:**
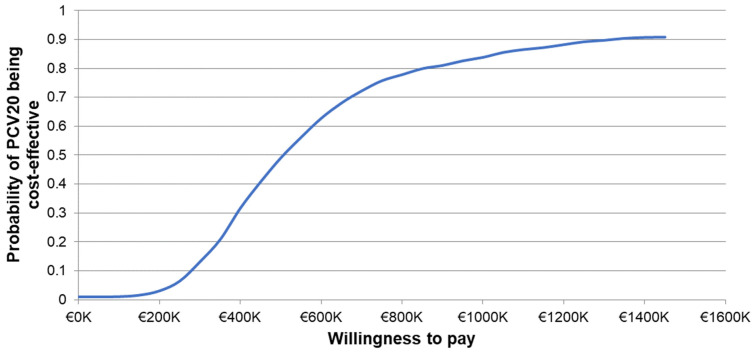
Cost-effectiveness acceptability curve of probabilistic sensitivity analysis comparing PCV20 versus V114. Abbreviations: PCV20 = 20-valent pneumococcal conjugate vaccine; V114 = 15-valent pneumococcal conjugate vaccine. Notes: The PSA tested the robustness of the model with respect to uncertainty in all input parameters with the exception of the vaccine prices, wherein a theoretical probability distribution was assigned to each parameter and a Monte Carlo simulation with 1000 iterations was performed by varying all parameters simultaneously. The curve depicts the probability of PCV20 (3 + 1) being cost-effective compared to V114 (2 + 1) based on the willingness-to-pay thresholds shown on the x-axis. Specifically, the probability was estimated based on the proportion of incremental cost-effectiveness ratios generated from the Monte Carlo simulation that were less than or equal to each willingness-to-pay threshold.

The one-way sensitivity analyses demonstrated similar sensitivity of the model’s results to individual inputs, with the ICERs for PCV20 compared to V114 in these analyses ranging from approximately EUR 320,000 to more than EUR 900,000 per QALY gained (Figure 2). The most sensitive model inputs included herd protection effects, serotype replacement, VE estimates, vaccine coverage rates, and the baseline incidence of meningitis. The results from the PSA showed that most of the ICERs for PCV20 were in the first quadrant, indicating both higher QALYs and higher costs with use of PCV20 compared to use of V114 (Figure 3). The cost-effectiveness acceptability curve showed that PCV20 had a 1% probability of being considered cost-effective compared to V114 at willingness-to-pay thresholds of EUR 50,000, EUR 100,000, and EUR 150,000 per QALY; the probability was 3% at a willingness-to-pay threshold of EUR 200,000 per QALY (Figure 4).

## 4. Discussion

The current study compared routine childhood immunization with PCV20 (3 + 1) to a currently recommended vaccination strategy, V114 (2 + 1), from a societal perspective in Germany. The analysis focused on V114 as a comparator instead of PCV13 because V114 is the most recently recommended PCV for routine childhood immunization in Germany [15]. The comparison between V114 (2 + 1) and PCV13 (2 + 1) was comprehensively assessed when V114 was evaluated for recommendation and reimbursement. In particular, V114 was projected to offer additional clinical benefits [13,14,57] at a parity price relative to PCV13 [51]. In the current analysis, the base-case results demonstrated that the small reductions in pneumococcal disease with the use of PCV20 came at a substantial cost (ICER of EUR 503,620 per QALY gained). The higher costs of PCV20 compared to V114 were largely attributable to higher vaccine acquisition and administration costs, given the need for an additional dose in the first year of life. The ICERs for PCV20 remained above EUR 180,000 per QALY in all the deterministic sensitivity analyses, which exceeded the current willingness-to-pay thresholds in most European countries [63]. The probability of PCV20 being cost-effective was 3% at a willingness-to-pay threshold of EUR 200,000 per QALY gained. Based on these findings, immunization with PCV20 (3 + 1) instead of V114 (2 + 1) may not be an economically efficient use of scarce healthcare resources in Germany.

While the base-case analysis predicted that the incorporation of PCV20 (3 + 1) into the routine childhood immunization program would result in fewer pneumococcal cases and deaths in Germany, these reductions in pneumococcal disease must be considered in the context of the increased costs associated with use of PCV20. Given that preventive health resources are limited in Germany and other countries, the use of additional resources for a childhood PCV program may result in fewer resources being available for other public health measures, which may include interventions that could have a greater impact on population health. In addition to economic considerations, the adoption of PCV20 in Germany should take into account VE uncertainties and potential implementation and logistical challenges. As previously discussed, there are significant uncertainties regarding the VE of PCV20, as several serotypes missed the co-primary endpoint of PPS non-inferiority based on the percentage of participants with pre-defined IgG levels in the two pivotal phase 3 trials [17]. Infants are vulnerable to infection and bear the highest incidence of IPD among all age groups, rendering the window between the last infant dose and the toddler dose a critical period for reducing the risk of IPD. Given the suboptimal PPS antibody responses of PCV20, it is unclear whether this vaccine will have effectiveness comparable to current PCVs among vaccinated individuals and maintain the herd protection of the general population established through use of current PCVs. In addition, recent shifts in IPD serotype distribution in Germany create additional uncertainties regarding the effectiveness of PCV20 for the prevention of pneumococcal disease. Specifically, the percentage of IPD cases caused by PCV20-non-V114 serotypes decreased from 29.7% in 2018–2019 to 15.7% in 2022–2023, while the proportion of cases caused by serotype 3 rose from 4.0% to 15.7% during this time period [31]. The inferior serum antibody response of PCV20 (3 + 1) to serotype 3 suggests that it may not be as effective in preventing IPD and other infections caused by this serotype. Because it would require an additional vaccine dose during infancy, the adoption of PCV20 would also increase the resources needed for the administration of PCVs at pediatric healthcare centers, and could lead to the need for additional healthcare visits and capacity for storage and waste disposal. These may add additional costs to the routine childhood immunization program. Moreover, there may be additional costs for caregivers, including transportation costs and productivity loss associated with additional child health visits. Admittedly, cost-sharing by manufacturers could reduce costs and enhance the cost-effectiveness of vaccines. However, information on manufacturer discounts is not publicly available, and it is beyond the scope of the current analysis. Overall, recommendations for the use of PCV20 for routine childhood immunization in Germany should consider the uncertain VE and indirect effects of PCV20, the higher costs of PCV20 compared to currently recommended PCVs, and the potential implementation and logistical challenges of switching back to a 3 + 1 PCV schedule.

Several prior CEAs compared the use of PCV20 (3 + 1) and V114 for routine childhood immunization. The findings from the current analysis are consistent with two CEAs of PCV20 (3 + 1) versus V114 (3 + 1) in the US [64,65]. Stoecker and colleagues from Tulane University and the US CDC estimated the ICER of PCV20 to be USD 153,715 per QALY gained [64], while Huang and colleagues from the manufacturer of V114 estimated the ICER of PCV20 to be USD 105,003 per QALY gained [65]. In both studies, the ICERs compared unfavorably with those of many other public health interventions. Rozenbaum and colleagues from the manufacturer of PCV20 similarly compared the use of PCV20 (3 + 1) versus V114 (3 + 1) for routine childhood immunization in the US, but instead concluded that PCV20 was the dominant strategy [66]. Moreover, using similar methods, Ta and colleagues conducted a recent CEA, sponsored by the manufacturer of PCV20, which compared the use of PCV20 (3 + 1) versus V114 (2 + 1) for routine childhood immunization in Germany, also concluding that PCV20 was the dominant strategy [21]. A summary of the three US CEAs performed by the US CDC highlighted the discrepancies between the model outcomes in the analysis conducted by Rozenbaum and colleagues and those in the other two models [65]. The summary also reviewed in detail the different inputs and assumptions regarding herd protection, which probably contributed to the varied conclusions across the models [65].

Several key methodological differences exist between the current analysis and the published CEA in Germany [21], which may have contributed to the substantial differences in the results. First, the VE inputs were estimated differently between the two studies. The current study applied serotype-specific VE and assumed reductions in the VE of PCV20 (3 + 1) against the serotypes that did not meet the statistical non-inferiority criteria in the clinical trial of PCV20 administered in a 3 + 1 schedule, with the scenario and one-way sensitivity analyses evaluating alternative VE inputs and assumptions, including no VE reduction for PCV20 and alternative VE inputs derived from modeling studies [57,58]. In contrast, Ta and colleagues applied the same VE to all serotypes, did not apply a VE reduction assumption to PCV20, and did not conduct sensitivity analyses, despite the uncertainty in the VE of PCV20 [21]. For VE against NBPP and pneumococcal AOM, Ta and colleagues applied PCV7 VE against all-cause pneumonia and all-cause AOM estimated from clinical trials conducted in the pre-PCV era [39,67,68], which reflected the serotype distribution at that time. It was unclear in this CEA whether or how the PCV7 VE was adjusted for current epidemiological data. Given that the proportions of these infections attributable to *S. pneumoniae* and caused by PCV7 serotypes are substantially lower than those prior to the introduction of PCVs [27,29], failure to adjust for these proportions would have led to considerably higher VE estimates. In addition, the indirect effects were also modeled differently between these two studies. The current study considered both herd protection and serotype replacement effects for IPD, using data from a German surveillance study [7], while Ta and colleagues assumed larger herd protection effects and did not consider serotype replacement effects. Indirect effects present an area of great uncertainty. The Committee for Medicinal Products for Human Use within the EMA noted that the herd protection effects of PCV20 were uncertain given the lower immunogenicity of this vaccine against certain serotypes [16]. Based on the results of the scenario analyses conducted in both studies, assumptions regarding indirect effects had a major impact on estimates of the ICER for PCV20 compared to V114. Moreover, not accounting for serotype replacement effects is likely to overestimate the benefits of higher-valency vaccines. The inputs used for the serotype epidemiology of IPD were another key difference between these studies, despite both analyses using data from the same source. The current study used the most recent data available (2022–2023) in the base case, while Ta and colleagues used data from prior to the COVID-19 pandemic (2018–2019) [21]. As shown in the present scenario analysis, using these earlier data substantially reduced the base-case ICER for PCV20 versus V114; thus, Ta and colleagues may have overestimated the impact of PCV20 on IPD by failing to account for the substantial shifts in serotype epidemiology that occurred in Germany during the COVID-19 pandemic.

The current study provides a rigorous and thorough economic evaluation of PCV20 (3 + 1) versus V114 (2 + 1) for routine childhood immunization in Germany. In particular, this CEA included extensive sensitivity analyses to evaluate the impact of the uncertainties over the VE and other model inputs, offering a comprehensive assessment of the cost-effectiveness of PCV20 versus V114. However, the results should be considered in the context of several limitations. First, the Markov model used in the current study is a static model, which may not be optimal for modeling the dynamics of diseases caused by a transmissible pathogen. In particular, indirect effects are incorporated in a static approach requiring inputs from observational studies, which may lead to high-level uncertainty. Second, both PCV20 and V114 were only recently approved for routine childhood immunization on the basis of vaccine immunogenicity data. Data regarding the effectiveness of these vaccines in preventing pneumococcal disease are lacking, and the VE estimates used in this analysis were derived from studies of PCV7 and PCV13 and required additional assumptions. For PCV20, the assumptions of a reduction in VE were based on the immunogenicity data in the phase 3 trial of PCV20 compared to PCV13. For V114, a modeling study predicts a higher VE for V114 than for PCV13 for serotype 3 in a 2 + 1 schedule [57]; however, the current study conservatively assumed the same VE values for serotype 3 between V114 and PCV13 in the base case. Additionally, there is substantial uncertainty in the inputs used for the indirect effects of PCV20 and V114, and with regard to the extent to which the serotype epidemiology of IPD will shift in Germany in the coming years. However, the assumptions for these parameters were evaluated in the sensitivity analyses and did not have a significant impact on the conclusions drawn from the model. Furthermore, this analysis did not account for vaccine use in adults, which would reduce the herd protection effects and probably increase the ICER for PCV20 compared to V114. In addition, the model did not consider vaccine adverse effects, although this is unlikely to have affected the results, given the similar frequencies of adverse effects associated with PCV20 and V114. Lastly, this analysis did not include all the potential costs that were anticipated to be associated with switching from a 2 + 1 to 3 + 1 schedule of PCV administration, although these would be anticipated to increase the ICER for PCV20 compared to V114.

## 5. Conclusions

Routine childhood immunization in Germany with PCV20 (3 + 1) is anticipated to result in small gains in QALYs and life years compared to V114 (2 + 1) in the base case. However, these benefits would come at a substantial cost, driven primarily by the higher costs of vaccine acquisition and administration with the use of PCV20. The projected ICERs for PCV20 compared to V114 in the model base case and sensitivity analyses varied substantially, highlighting the impact of uncertainties regarding the VE and the indirect effects of PCV20. However, all the ICERs for PCV20 exceeded common willingness-to-pay thresholds cited in the literature, indicating that immunization with PCV20 (3 + 1) instead of V114 (2 + 1) may not be an economically efficient use of healthcare resources in Germany. Policy and practice decisions regarding use of PCV20 for routine childhood immunization in Germany should consider the lower serum antibody response of PCV20 in certain serotypes and the higher costs and logistical challenges associated with administering PCV20 in a 3 + 1 schedule.

## Figures and Tables

**Table 1:** Epidemiological inputs in the base-case analysis.

Parameters	Input Value						
	Age Group (Years)						
	0–1	2–4	5–15	16–49	50–59	60–69	70–100
***Pneumococcal disease annual incidence rates (cases per 100,000)***							
IPD ^a^	11.04	3.69	1.76	1.69	8.76	14.47	24.77
IPD—meningitis (%)	27.08	20.00	65.91	19.82	14.07	10.09	3.98
IPD—bacteremia without a focus (%)	60.42	52.00	15.91	27.93	34.67	31.65	29.65
IPD—bacteremic pneumonia (%)	12.50	28.00	18.18	52.25	51.26	58.26	66.37
NBPP ^b^							
Inpatient NBPP	109.14	62.67	13.53	10.25	33.63	84.13	290.05
Outpatient NBPP	221.02	296.16	82.15	36.92	60.78	69.74	102.99
Pneumococcal AOM ^c^							
Simple AOM	950.27	1115.92	272.88	NA	NA	NA	NA
Recurrent AOM	200.80	252.67	26.36	NA	NA	NA	NA
AOM with TT placement	181.04	414.28	165.18	NA	NA	NA	NA
***Case fatality rates***							
Meningitis ^d^	0.0980	0.0980	0.0980	0.0874	0.1316	0.1250	0.2229
Bacteremia without ^a^ focus/bacteremic pneumonia ^d^	0.0200	0.0200	0.0200	0.0874	0.1316	0.1250	0.2229
Inpatient NBPP ^e^	0.0022	0.0013	0.0038	0.0442	0.1095	0.1410	0.2036

Abbreviations: IPD = invasive pneumococcal disease; NBPP = non-bacteremic pneumococcal pneumonia; AOM = acute otitis media; TT = tympanostomy tube; NA = non-applicable. Notes: ^a^ Annual incidence rates of IPD were obtained from Weaver et al., 2024 [26] for individuals < 16 years of age and from Deb et al., 2022 [25] for individuals 16 years of age or older. ^b^ Annual incidence rates of NBPP were estimated by multiplying annual incidence rates of all-cause pneumonia and the proportion of pneumonia cases attributable to *S. pneumoniae*. Annual incidence rates of all-cause pneumonia were obtained from the same studies as IPD [25,26]. The proportion of pneumonia cases attributable to *S. pneumoniae* was estimated to be 9.2% based on data from Ieven et al., 2018 [27]. ^c^ Annual incidence rates of pneumococcal AOM were estimated by multiplying annual incidence rates of all-cause AOM and the proportion of AOM cases attributable to *S. pneumoniae*, and were applied only to individuals < 16 years of age. Annual incidence rates of all-cause AOM were obtained from Hu et al., 2022 [28]. The proportion of AOM cases attributable to *S. pneumoniae* was estimated to be 6.61% based on data from Imöhl et al., 2018 [29]. ^d^ The case fatality rates for IPD were obtained from von Kries et al., 2000 for individuals < 16 years of age [30] and from Deb et al., 2022 for individuals 16 years of age or older [25]. ^e^ The case fatality rates for inpatient NBPP were obtained from the estimated case fatality rates for inpatient pneumonia in the studies conducted by Weaver et al., 2024 for individuals < 16 years of age [26] and Deb et al., 2022 for individuals 16 years of age or older [25].

**Table 2:** Percentages of pneumococcal disease caused by PCV20-unique serotypes by age group in the base-case analysis.

PCV20 Non-V114 Serotypes (%)	Age Group (Years)						
	0–1	2–4	5–15	16–49	50–59	60–69	70–100
IPD ^a^	14.9	4.5	10.6	8.1	6.6	7.6	7.6
NBPP ^b^	14.9	4.5	10.6	8.1	6.6	7.6	7.6
Pneumococcal AOM ^c^	9.8	9.8	9.8	NA	NA	NA	NA

Abbreviations: IPD = invasive pneumococcal disease; NBPP = non-bacteremic pneumococcal pneumonia; AOM = acute otitis media; NA = not applicable. Notes: ^a^ The serotype distribution for IPD was obtained from a surveillance study conducted in Germany by van der Linden M and Itzek A (data on file, Merck & Co., Inc.) [31]. The most recent data available (2022–2023) were used in the base case. ^b^ The serotype distribution for NBPP was assumed to be the same as that of IPD. ^c^ The serotype distribution for pneumococcal AOM was obtained from an observational study in Germany conducted by Imöhl et al., 2021 [29]. The same serotype distribution was applied to simple AOM, recurrent AOM, and AOM with tympanostomy tube placement.

**Table 3:** Vaccine effectiveness of V114 (2 + 1) and PCV20 (3 + 1) in the base-case analysis.

Disease	Vaccine Product	Serotype-Specific VE																			
**PPS**	**Serotype**	**PCV13**													**V114-non-PCV13**		**PCV20-non-V114**				
		**1**	**3**	**4**	**5**	**6A**	**6B**	**7F**	**9V**	**14**	**18C**	**19A**	**19F**	**23F**	**22F**	**33F**	**8**	**10A**	**11A**	**12F**	**15B**
**IPD ^a^**	V114	85.1	64.5	93.0	79.0	92.7	92.7	91.4	97.3	96.8	93.0	83.2	84.4	93.0	84.2	84.2					
	PCV20 ^b^	65.3	40.0	69.8	87.0	86.0	94.0	97.0	75.0	94.0	97.0	86.0	87.0	73.5	86.0	86.0	86.0	86.0	86.0	43.0	86.0
**NBPP ^c^**	V114	77.0	77.0	77.0	77.0	77.0	77.0	77.0	77.0	77.0	77.0	77.0	77.0	77.0	77.0	77.0					
	PCV20 ^b^	57.8	38.5	57.8	77.0	77.0	77.0	77.0	57.8	77.0	77.0	77.0	77.0	57.8	77.0	77.0	77.0	77.0	77.0	38.5	77.0
**Pneumococcal AOM (simple or recurrent) ^d^**	V114	86.0	15.0	57.0	86.0	100.0	57.0	86.0	57.0	57.0	57.0	91.0	57.0	57.0	57.0	57.0					
	PCV20 ^b^	64.5	7.5	42.8	86.0	100.0	57.0	86.0	42.8	57.0	57.0	91.0	57.0	42.8	57.0	57.0	57.0	57.0	57.0	28.5	57.0
**Pneumococcal AOM (with TT placement) ^e^**	V114	68.8	68.8	68.8	68.8	68.8	68.8	68.8	68.8	68.8	68.8	68.8	68.8	68.8	68.8	68.8					
	PCV20 ^b^	51.6	34.4	51.6	68.8	68.8	68.8	68.8	51.6	68.8	68.8	68.8	68.8	51.6	68.8	68.8	68.8	68.8	68.8	34.4	68.8
**PTD**	**Serotype**	**PCV13**													**V114-non-PCV13**		**PCV20-non-V114**				
		**1**	**3**	**4**	**5**	**6A**	**6B**	**7F**	**9V**	**14**	**18C**	**19A**	**19F**	**23F**	**22F**	**33F**	**8**	**10A**	**11A**	**12F**	**15B**
**IPD ^a,f^**	V114	87.0	80.0	96.1	87.0	95.9	95.9	97.0	100.0	97.6	97.0	92.0	92.3	98.0	86.0	86.0					
	PCV20	87.0	80.0	96.1	87.0	95.9	95.9	97.0	100.0	97.6	97.0	92.0	92.3	98.0	86.0	86.0	86.0	86.0	86.0	86.0	86.0
**NBPP ^c^**	V114	77.0	77.0	77.0	77.0	77.0	77.0	77.0	77.0	77.0	77.0	77.0	77.0	77.0	77.0	77.0					
	PCV20	77.0	77.0	77.0	77.0	77.0	77.0	77.0	77.0	77.0	77.0	77.0	77.0	77.0	77.0	77.0	77.0	77.0	77.0	77.0	77.0
**Pneumococcal AOM(Simple or recurrent AOM) ^d^**	V114	86.0	15.0	57.0	86.0	100.0	57.0	86.0	57.0	57.0	57.0	91.0	57.0	57.0	57.0	57.0					
	PCV20	86.0	15.0	57.0	86.0	100.0	57.0	86.0	57.0	57.0	57.0	91.0	57.0	57.0	57.0	57.0	57.0	57.0	57.0	57.0	57.0
**Pneumococcal AOM(with TT placement) ^e^**	V114	68.8	68.8	68.8	68.8	68.8	68.8	68.8	68.8	68.8	68.8	68.8	68.8	68.8	68.8	68.8					
	PCV20	68.8	68.8	68.8	68.8	68.8	68.8	68.8	68.8	68.8	68.8	68.8	68.8	68.8	68.8	68.8	68.8	68.8	68.8	68.8	68.8

Abbreviations: VE = vaccine effectiveness; PCV7 = 7-valent pneumococcal conjugate vaccine; PCV13 = 13-valent pneumococcal conjugate vaccine; V114 = 15-valent pneumococcal conjugate vaccine; PCV20 = 20-valent pneumococcal conjugate vaccine; PPS = post-primary series; IPD = invasive pneumococcal disease; NBPP = non-bacteremic pneumococcal pneumonia; AOM = acute otitis media; TT = tympanostomy tube; PTD = post-toddler dose. Notes: ^a^ The VE against IPD for V114 was derived from an observational study of PCV13 (2 + 1) conducted in Europe by Savulescu et al., 2022 [33]. The VE against IPD for PCV20 was estimated based on the VE for PCV7 serotypes in a case–control study of PCV7 (3 + 1) conducted by Whitney et al., 2006 [34] and a case-control study of PCV13 (3 + 1) by Moore et al., 2016 [35]. The VE values for the serotypes in V114 and PCV20 that are not included in PCV13 were assumed to be equal to the average VE of PCV13 against the shared 13 serotypes. ^b^ In the base case, PPS VE reductions were assumed for the serotypes in PCV20 that did not meet the noninferiority criterion in the clinical trial of PCV20 administered in a 3 + 1 schedule (B7471011). Specifically, for all pneumococcal disease categories and during the first year of life only, 50% VE reduction was assumed for serotype 3 and 25% VE reduction was assumed for serotypes 1, 4, 9V, 12F, and 23F. ^c^ The VE against inpatient and outpatient NBPP was obtained from a surveillance study in southern Israel by Lewnard et al., 2021 [36] and was applied for all serotypes. ^d^ The VE against pneumococcal AOM was obtained from a clinical trial of PCV7 conducted by Eskola et al., 2001 [37] and an observational study conducted by Pichichero et al., 2018 [38]. The VE for the serotypes in V114 and PCV20 that are not included in PCV13 was assumed to be the same as the average VE for PCV7 against vaccine serotypes reported by Eskola et al., 2001 [37]. ^e^ For all serotypes, the VE against pneumococcal AOM with TT placement was estimated from a 20.3% VE against all-cause AOM with TT placement reported in a clinical trial of PCV7 (3 + 1) [39], adjusted for the estimated proportion of all-cause AOM attributable to *S. pneumoniae* (44%) [40] and the proportion of pneumococcal pneumonia cases accounted for by PCV7 serotypes estimated by a study conducted in the United States in the pre-vaccination era (67.1%) [41]. ^f^ The PTD serotype-specific VE against IPD for V114 and PCV20 was assumed to be the same; for each serotype, the larger of the VE estimates obtained for V114 and PCV20 was used for both vaccines.

**Table 4:** Utility inputs in the base-case analysis.

Parameters	Input Value						
	Age Group (Years)						
	0–24	25–34	35–45	45–54	55–64	65–74	75–100
***Health state utility values***							
Baseline (general population without pneumococcal disease or PMS) ^a^	0.972	0.973	0.966	0.945	0.922	0.891	0.839
PMS, neurological deficits ^b^	0.68	0.68	0.68	0.68	0.68	0.68	0.68
PMS, hearing loss ^b^	0.73	0.73	0.73	0.73	0.73	0.73	0.73
	**0–15**	**16–100**					
***QALY decrements related to each pneumococcal disease episode ^c^***							
Meningitis	0.023	0.071					
Bacteremia without a focus	0.008	0.071					
Bacteremic pneumonia	0.008	0.071					
Inpatient NBPP	0.006	0.071					
Outpatient NBPP	0.004	0.005					
Simple pneumococcal AOM	0.005	NA					
Recurrent pneumococcal AOM	0.005	NA					
AOM with TT placement	0.005	NA					

Abbreviations: PMS = post-meningitis sequelae; QALY = quality-adjusted life year; NBPP = non-bacteremic pneumococcal pneumonia; AOM = acute otitis media; TT = tympanostomy tube. Notes: ^a^ Baseline utility values for individuals without pneumococcal disease were sourced from age-specific utilities of the German general population reported in Szende et al., 2014 [49]. ^b^ Health state utilities for PMS were obtained from Rubin, et al., 2010 [47]. ^c^ QALY decrements per episode of IPD, NBPP, and pneumococcal AOM were obtained from Rubin et al., 2010 for individuals < 16 years of age [47] and from Mangen et al., 2015 for individuals 16 years of age or older [48].

**Table 5:** Cost inputs in the base-case analysis.

Parameters		Input Value								
***Vaccine Costs (in 2023 EUR)***										
Vaccine acquisition costs (per dose) ^a^										
V114	59.80									
PCV20	66.95									
Vaccine administration costs (per dose) ^b^										
V114	8.00									
PCV20	8.00									
	**Age group (years)**									
	**0–15**	**16–100**								
***Costs per pneumococcal disease episode (in 2023 EUR)***										
Direct medical costs ^c^										
Meningitis	6464	8584								
Bacteremia without focus	6464	8584								
Bacteremic pneumonia	6464	8584								
Inpatient NBPP	2841	6614								
Outpatient NBPP	71	58								
Simple pneumococcal AOM	61	NA								
Recurrent pneumococcal AOM	69	NA								
AOM with TT placement	443	NA								
	**Age group (years)**									
	**0–4**	**5–15**	**15–20**	**20–25**	**25–30**	**30–40**	**40–55**	**55–60**	**60–65**	**65–100**
Indirect costs ^d^										
Meningitis	1875	1875	527	1316	1543	1585	1628	1558	1226	169
Bacteremia without a focus	919	919	258	645	756	777	798	764	601	83
Bacteremic pneumonia	919	919	258	645	756	777	798	764	601	83
Inpatient NBPP	1221	1221	343	857	1005	1033	1061	1015	799	110
Outpatient NBPP	473	473	133	332	389	400	411	393	309	43
Simple pneumococcal AOM	473	473	NA	NA	NA	NA	NA	NA	NA	NA
Recurrent pneumococcal AOM	473	473	NA	NA	NA	NA	NA	NA	NA	NA
AOM with TT placement	473	473	NA	NA	NA	NA	NA	NA	NA	NA
***Annual direct costs of PMS(in 2023 EUR)***										
Direct medical and non-medical costs of PMS ^e^										
PMS, neurological deficits	1694	1694	1694	1694	1694	1694	1694	1694	1694	1694
PMS, hearing loss	2974	2974	2974	2974	2974	2974	2974	2974	2974	2974
Average annual earnings ^f^	NA	NA	14,577	36,417	42,694	43,861	45,046	43,109	33,927	4669

Abbreviations: V114 = 15-valent pneumococcal conjugate vaccine; PCV20 = 20-valent pneumococcal conjugate vaccine; NBPP = non-bacteremic pneumococcal pneumonia; AOM = acute otitis media; TT = tympanostomy tube; PMS = post-meningitis sequelae; NA = non-applicable. Notes: ^a^ Vaccine acquisition costs for V114 and PCV20 were obtained from the BAPO^®^ LAUER-Taxe Pricing database [51]. ^b^ Vaccine administration costs were estimated based on the average reimbursement fee in a sample of German vaccination agreements between the statutory health insurance system and the National Association of Statutory Health Insurance Physicians. ^c^ Direct medical costs for each pneumococcal disease episode were obtained from Hu et al., 2023 for individuals < 16 years of age [52] and from Deb et al., 2022 for individuals 16 years of age or older [25]. The costs for all-cause pneumonia and AOM were used as model inputs due to lack of cost data specifically for NBPP and pneumococcal AOM. ^d^ Indirect costs associated with each episode of pneumococcal disease included lost productivity experienced by patients or caregivers. These costs were estimated using data on missed workdays from Li et al., 2023 [53], and the average annual earnings in each age group were estimated using data from the Federal Statistical Office of Germany [54,55]. The costs for all-cause pneumonia and AOM were used as model inputs due to lack of cost data specifically for NBPP and pneumococcal AOM. ^e^ Annual direct medical and non-medical costs associated with PMS were obtained from Talbird et al., 2010 [56], and were applied to all age groups. ^f^ Average annual earnings, calculated by multiplying the average gross earnings by the labor force participation rate for each age group [54,55], were used to estimate productivity loss associated with pneumococcal disease, PMS, and premature death.

**Table 6:** Base case results.

Outcomes	PCV20 (3 + 1 Schedule)	V114 (2 + 1 Schedule)	Incremental Outcomes (PCV20 versus V114)
**Cost outcomes (in 2023 EUR, discounted)**			
Vaccine acquisition costs	160,757,092	111,144,118	49,612,973
Vaccine administration costs	19,209,212	14,868,778	4,340,433
IPD treatment costs	6,984,679	7,232,406	−247,727
NBPP treatment costs	41,650,604	42,032,280	−381,676
Pneumococcal AOM treatment costs	9,059,576	9,283,841	−224,266
PMS treatment costs	1,370,853	1,496,730	−125,876
Indirect costs	55,157,026	57,861,839	−2,704,813
Costs of premature death	131,785,723	133,696,348	−1,910,625
**Total Costs**	425,974,765	377,616,341	48,358,424
**Clinical outcomes (undiscounted)**			
IPD cases	4659	4745	−86
NBPP cases	82,521	83,194	−672
Pneumococcal AOM cases	75,888	78,107	−2219
PMS cases	65	68	−3
IPD deaths	790	800	−10
NBPP deaths	7064	7065	0
**QALYs and life years (discounted)**			
Total QALYs	21,556,792	21,556,696	96
Total life years	22,448,853	22,448,779	75
**ICERs**			
Cost per QALY gained			503,620
Cost per life year gained			648,546

Abbreviations: PCV20 = 20-valent pneumococcal conjugate vaccine; V114 = 15-valent pneumococcal conjugate vaccine; IPD = invasive pneumococcal disease; AOM = acute otitis media; PMS = post-meningitis sequelae; NBPP = non-bacteremic pneumococcal pneumonia; QALY = quality-adjusted life year; ICER = incremental cost-effectiveness ratio.

**Table 7:** Incremental costs and effectiveness outcomes comparing PCV20 to V114 in scenario analyses.

Scenario	Assumptions or Inputs in the Base Case	Assumptions or Inputs in the Scenario Analysis	Incremental Costs (in 2023 EUR)	Incremental QALYs Gained	ICER for PCV20 vs. V114 (Cost per QALY Gained, in 2023 EUR)
1. Time horizon	Lifetime	10 years	52,041,409	19	2,672,457
2. Model perspective	Societal perspective (including vaccine acquisition and administration costs, direct medical costs, direct non-medical costs, and indirect costs)	Healthcare sector perspective (only including vaccine acquisition and administration costs and direct medical costs)	52,973,862	96	551,687
3. Target population	A single birth cohort	Entire German population	1,278,538,860	6986	183,006
4. Target population and time horizon	Target population: a single birth cohort Time horizon: lifetime	Target population: entire German population Time horizon: 10 years	429,468,264	528	813,056
5. IPD serotype distribution	Use of most recent German data (2022–2023) from van der Linden et al., 2024 [31]	Use of data from prior to the COVID-19 pandemic (2018–2019) from van der Linden et al., 2024 [31]	41,828,610	199	210,403
6. Proportion of pneumonia and AOM cases attributable to *S. pneumoniae*	Inputs from studies conducted in Germany	Inputs based on recent US data presented at the CDC ACIP meetings by King et al. [61] ^a^	46,926,936	110	424,805
7. PCV20 VE reduction	Assuming reductions in PPS VE for the six serotypes that did not meet the statistical non-inferiority criterion in the PCV20 (3 + 1) clinical trial (serotypes 1, 3, 4, 9V, 12F, and 23F) in all pneumococcal disease categories	No VE reductions assumed ^b^	46,978,220	114	412,159
8. IPD and NBPP VE	VE against IPD: VE of V114 was derived from an observational study of PCV13 (2 + 1); VE of PCV20 was derived from PCV7 (3 + 1) and PCV13 (3 + 1) case–control studies VE against NBPP: For both V114 and PCV20, VE was obtained from a surveillance study conducted in southern Israel by Lewnard et al., 2021 [36]	VE against IPD: For both V114 and PCV20, VE was obtained from modeling predictions for V114 (2 + 1) and PCV20 (3 + 1) by Ryman et al., 2024 [57,58] VE against NBPP: For both V114 and PCV20, VE was obtained from an observational cohort study conducted in China by Zhang et al., 2021 [59]	49,749,027	82	606,835
9. Waning of vaccine effects	Full VE for first five years after vaccination followed by a linear reduction to 0% over the next five years	Full VE for the first 10 years after vaccination and no VE thereafter	48,293,570	97	499,005
10. Indirect effects	Herd protection effects and serotype replacement from a study conducted in Germany by van der Linden et al., 2015 [8] ^c^	Herd protection effects and no serotype replacement based on a CEA study conducted in the US by Stoecker et al., 2013 [60] ^c^	46,729,650	158	295,248
11. Re-emergence of IPD with PCV20 (3 + 1)	No re-emergence of IPD caused by serotypes included in PCV13	Re-emergence of IPD caused by serotypes shared between PCV13 and PCV20 for which PCV20 was assumed to have reduced VE (serotypes 1, 4, 9V, and 23F) ^d^	78,477,554	−765	PCV20 is dominated by V114
12. Utility inputs	Inputs for QALY decrements for pneumococcal disease were sourced from Rubin et al., 2010 [47] and Mangen et al., 2015 [48]	Inputs for QALY decrements for pneumococcal disease were sourced from a pooled analysis performed by Tang et al., 2022 [62]	48,358,424	93	518,169
13. Vaccine coverage rate	Different vaccine coverage rates for 3 + 1 and 2 + 1 schedules were applied, based on a German study conducted by Laurenz et al., 2021 [32]	Vaccine coverage rates for PCV20 (3 + 1) were assumed to be the same as those for V114 (2 + 1)	55,762,365	112	500,069

Abbreviations: PCV20 = 20-valent pneumococcal conjugate vaccine; V114 = 15-valent pneumococcal conjugate vaccine; PCV13 = 13-valent pneumococcal conjugate vaccine; PCV7 = 7-valent pneumococcal conjugate vaccine; VE = vaccine effectiveness; IPD = invasive pneumococcal disease; NBPP = non-bacteremic pneumococcal pneumonia; QALY = quality-adjusted life year; ICER = incremental cost-effectiveness ratio; US = United States. Notes: ^a^ The proportion of pneumonia cases attributable to *S. pneumoniae* was 14.0% and the proportion of AOM cases attributable to *S. pneumoniae* was 11.8%. Data were presented during a US Centers for Disease Control and Prevention Advisory Committee on Immunization Practices meeting in 2023 [61]. ^b^ In this scenario, without the assumption of PPS VE reductions for serotypes 1, 3, 4, 9V, 12F and 23F, the VE values of PCV20 against IPD were 87.0% for serotype 1, 80.0% for serotype 3, 93.0% for serotype 4, 100.0% for serotype 9V, 98.0% for serotype 23F, and 86.0% for serotype 12F; the VE of PCV20 against NBPP was 77.7% for all six serotypes; the VE values of PCV20 against simple or recurrent pneumococcal AOM were 86.0% for serotype 1, 15% for serotype 3, and 47% for serotypes 4, 9V, 23F, and 12F; and the VE of PCV20 against AOM with tympanostomy tube placement was 68.8% for all six serotypes. ^c^ In the base case, the maximum herd protection effects were a 70.0% reduction in pneumococcal disease among individuals < 16 years of age and a 42.2% reduction for individuals 16 years of age or older, with full herd protection effects reached five years after vaccine introduction. Serotype replacement was also considered based on German surveillance data reported by van der Linden et al., 2015 [8]. In the alternative scenario analysis, the herd protection effect was assumed to be a 7.8% reduction each year, with a maximum effect of 33.4% reached in the 5th year after vaccine introduction, and no serotype replacement was assumed, based on the cost-effectiveness analysis conducted in the US by Stoecker et al., 2013 [60], which used data from the Active Bacterial Core surveillance at the US Centers for Disease Control and Prevention for the herd protection and serotype replacement inputs. ^d^ Re-emergence of serotype 3 was not considered in the scenario analysis. Because the incidence rate of IPD caused by serotype 3 did not decline in German children after PCV13 introduction (van der Linden et al., 2015 [8]), the model assumed that the baseline incidence rates used in the model reflected the low VE of PCV13 against this serotype and did not consider a further increase in the incidence of IPD caused by this serotype as a result of use of PCV20.

## Data Availability

The data used in this study are presented in the article or the Supplementary Materials.

## Supplementary Materials

The following supporting information can be downloaded at: https://www.mdpi.com/article/10.3390/vaccines12091045/s1, Table S1: Vaccine effectiveness of V114 (2 + 1) and PCV20 (3 + 1) against IPD and NBPP in the scenario analysis with alternative IPD and NBPP VE; Table S2: Percentages of disease caused by PCV20-unique serotypes by age group in the scenario analysis with an alternative serotype distribution for IPD; Table S3: QALY decrements for IPD, NBPP, and pneumococcal AOM in the scenario analysis with alternative utility inputs.
