# Supplementary material for: Cost-Effectiveness Analysis of Routine Childhood Immunization with 20-Valent versus 15-Valent Pneumococcal Conjugate Vaccines in Germany
Source: Vaccines (Basel). 2024 Sep 12;12(9):1045. doi: 10.3390/vaccines12091045 (PMC11435687; doi:10.3390/vaccines12091045)
Supplement: Supplementary file 1 [file vaccines-12-01045-s001.zip › vaccines-3172986-supplementary.pdf]

Supplementary Documents for

Cost-Effectiveness Analysis of Routine Childhood Immunization with 20-Valent versus 15-Valent Pneumococcal Conjugate Vaccines in Germany

Min Huang [1,*], Jessica P. Weaver [1], Elamin Elbasha [1], Thomas Weiss [1], Natalie Banniettis [1], Kristen Feemster [1], Meghan White [1] and Matthew S. Kelly [2]

[1] Merck Research Laboratory, Merck & Co., Inc., Rahway, NJ 07065, USA
[2] Division of Pediatric Infectious Diseases, Duke University School of Medicine, Durham, NC 27710, USA
* Correspondence: min_huang@merck.com; Tel.: +1-215-652-5974

Supplementary Table S1. Vaccine effectiveness of V114 (2 +1) and PCV20 (3 +1) against IPD and NBPP in the scenario analysis with alternative IPD and NBPP VE

Disease	Vaccine product	Serotype-specific VE																			
**PPS**	**Serotype**	**PCV13**													**V114-non-PCV13**		**PCV20-non-V114**				
		**1**	**3**	**4**	**5**	**6A**	**6B**	**7F**	**9V**	**14**	**18C**	**19A**	**19F**	**23F**	**22F**	**33F**	**8**	**10A**	**11A**	**12F**	**15B**
**IPD** [a]	V114	88.0	93.0	97.0	79.0	88.0	83.0	91.0	98.0	99.0	97.0	78.0	81.0	100.0	84.2	84.2					
	PCV20	67.0	64.0	77.0	79.0	74.0	83.0	92.0	99.0	75.0	89.0	80.0	96.0	93.0	86.0	86.0	86.0	86.0	86.0	64.5	86.0
**NBPP** [b]	V114	62.1	62.1	62.1	62.1	62.1	62.1	62.1	62.1	62.1	62.1	62.1	62.1	62.1	62.1	62.1					
	PCV20 [c]	46.6	31.1	46.6	62.1	62.1	62.1	62.1	46.6	62.1	62.1	62.1	62.1	46.6	62.1	62.1	62.1	62.1	62.1	31.1	62.1
**PTD**	**Serotype**	**PCV13**													**V114-non-PCV13**		**PCV20-non-V114**				
		**1**	**3**	**4**	**5**	**6A**	**6B**	**7F**	**9V**	**14**	**18C**	**19A**	**19F**	**23F**	**22F**	**33F**	**8**	**10A**	**11A**	**12F**	**15B**
**IPD** [a,d]	V114	88.0	93.0	97.0	79.0	88.0	74.0	92.0	99.0	99.0	97.0	84.0	96.0	100.0	86.0	86.0					
	PCV20	88.0	93.0	97.0	79.0	88.0	74.0	92.0	99.0	99.0	97.0	84.0	96.0	100.0	86.0	86.0	86.0	86.0	86.0	86.0	86.0
**NBPP** [b]	V114	62.1	62.1	62.1	62.1	62.1	62.1	62.1	62.1	62.1	62.1	62.1	62.1	62.1	62.1	62.1					
	PCV20	62.1	62.1	62.1	62.1	62.1	62.1	62.1	62.1	62.1	62.1	62.1	62.1	62.1	62.1	62.1	62.1	62.1	62.1	62.1	62.1

Abbreviations: VE = vaccine effectiveness; V114 = 15-valent pneumococcal conjugate vaccine; PCV20 = 20-valent pneumococcal conjugate vaccine; IPD = invasive pneumococcal disease; NBPP = non-bacteremic pneumococcal pneumonia; PCV13 = 13-valent pneumococcal conjugate vaccine.

Notes:

a. The VE estimates against IPD for V114 and PCV20 were derived from two modeling studies conducted by Ryman et al., 2024. The VE for V114 was from the study that modeled administration in a 2+1 schedule and the VE for PCV20 was from the study that modeled administration in a 3+1 schedule. The VE for the serotypes in V114 and PCV20 that are not included in PCV13 was assumed to be equal to the average VE of PCV13 against the shared 13 serotypes.
b. The VE against inpatient and outpatient NBPP was obtained from an observational study conducted in China by Zhang et al., 2021, and was applied for all serotypes.
c. For NBPP, PPS VE reductions were assumed for the serotypes in PCV20 that did not meet the noninferiority criterion in the clinical trial of PCV20 administered to children in a 3+1 schedule (B7471011). Specifically, a 50% reduction in the VE was assumed for serotype 3 and a 25% reduction in the VE was assumed for serotypes 1, 4, 9V, 12F, and 23F.
d. The PTD serotype-specific VE against IPD for V114 and PCV20 was assumed to be the same; for each serotype, the larger of the VE estimates obtained for V114 and PCV20 was used for both vaccines.

Supplementary Table S2. Percentages of disease caused by PCV20-unique serotypes by age group in the scenario analysis with an alternative serotype distribution for IPD

Serotypes in PCV20 not V114 (%)	Age group (years)						
	**0-2**	**2-4**	**5-15**	**16-49**	**50-59**	**60-69**	**70-100**
IPD [a]	29.7	33.3	29.7	34.3	31.3	24.4	24.4
NBPP [b]	29.7	33.3	29.7	34.3	31.3	24.4	24.4
Pneumococcal AOM [c]	9.8	9.8	9.8	NA	NA	NA	NA

Abbreviations: IPD = invasive pneumococcal disease; NBPP = non-bacteremic pneumococcal pneumonia; AOM = acute otitis media; NA = not applicable.

Notes:

a. The serotype distribution for IPD was obtained from a surveillance study conducted in Germany by van der Linden M and Itzek A (data on file, Merck & Co.). Data from 2018-2019 were used in this scenario analysis.
b. The serotype distribution for NBPP was assumed to be the same as for IPD.
c. The serotype distribution for pneumococcal AOM was obtained from an observational study in Germany conducted by Imöhl et al. 2021, and was the same serotype distribution used in the base case. The same serotype distribution was applied to simple AOM, recurrent AOM, and AOM with tympanostomy tube placement.

Supplementary Table S3. QALY decrements for IPD, NBPP, and pneumococcal AOM in the scenario analysis with alternative utility inputs [a]

Age group (years)	QALY decrements for IPD			QALY decrements for NBPP		QALY decrements for AOM		
	Meningitis	Bacteremia without a focus	Bacteremic pneumonia	Inpatient	Outpatient	Simple AOM	Recurrent AOM	AOM with TT placement
0 – 17	0.0165	0.0016	0.0016	0.0105	0.0004	0.0016	0.0016	0.0016
≥18	0.0547	0.0547	0.0547	0.0396	0.0094	NA	NA	NA

Abbreviations: QALY=quality-adjusted life years; IPD = invasive pneumococcal disease; NBPP = non-bacteremic pneumococcal pneumonia; AOM = acute otitis media; TT = tympanostomy tube; NA= non-applicable.

Notes:

a. QALY decrements per episode for each pneumococcal disease were derived from a meta-analysis conducted by Tang et al., 2022.
